# Genome Editing in Sugarcane: Challenges Ahead

**DOI:** 10.3389/fpls.2016.01542

**Published:** 2016-10-13

**Authors:** Chakravarthi Mohan

**Affiliations:** Molecular Biology Laboratory, Department of Genetics and Evolution, Federal University of Sao CarlosSao Carlos, Brazil

**Keywords:** CRISPR/Cas9, genome editing, polyploid, sugarcane, TALENs, ZFNs

## Abstract

Genome editing opens new and unique opportunities for researchers to enhance crop production. Until 2013, the zinc finger nucleases (ZFNs) and transcription activator-like effector nucleases (TALENs) were the key tools used for genome editing applications. The advent of RNA-guided engineered nucleases - the type II clustered regularly interspaced short palindromic repeat (CRISPR)/Cas9 (CRISPR-associated) system from *Streptococcus pyogenes* holds great potential since it is simple, effective and more versatile than ZFNs and TALENs. CRISPR/Cas9 system has already been successfully employed in several crop plants. Use of these techniques is in its infant stage in sugarcane. Jung and Altpeter (2016) have reported TALEN mediated approach for the first time to reduce lignin content in sugarcane to make it amenable for biofuel production. This is so far the only report describing genome editing in sugarcane. Large genome size, polyploidy, low transformation efficiency, transgene silencing and lack of high throughput screening techniques are certainly great challenges for genome editing in sugarcane which would be discussed in detail in this review.

## Introduction

Sugarcane is an economically important crop and is cultivated throughout the world for its sugar and ethanol. Brazil is the world’s largest producer of sugarcane followed by India, China, Thailand, Pakistan, and Mexico (FAOSTAT, 2015). It is among the most highly photosynthetically efficient C_4_ crops and can accumulate high quantities of biomass thereby making it a potential bio energy feedstock. The modern sugarcane varieties are derived from interspecific hybridization between *Saccharum officinarum* and *Saccharum spontaneum*, resulting in highly polyploid and aneuploid plants with chromosomes ranging from 80 to 120. Typically, modern sugarcane presents >8 homologous copies of each chromosome from *S. officinarum* and several copies from *S. spontaneum*. This complex nature has hindered advances in marker assisted breeding of the crop.

Since the first genetic engineered sugarcane by Bower and Birch (1992), an array of methodologies have been adopted and now several research groups from all over the world have well established protocols for developing genetically modified (GM) sugarcane and several genes have been transformed in sugarcane, despite variable success in transgene integration and expression. GM sugarcane has been approved in Indonesia for commercial cultivation (Parisi et al., 2016) and is in the pipeline in several other countries. However, several challenges such as transgene inactivation, low transformation efficiency and time constraints deter sugarcane improvement through genetic engineering (Hansom et al., 1999; Joyce et al., 2010).

Genome or gene editing (GE) is a type of genetic modification in which DNA is inserted, deleted or replaced in the genome of an organism using engineered nucleases. These nucleases create site-specific double-strand breaks (DSBs) at desired locations in the genome. The induced DSBs are repaired through non-homologous end joining (NHEJ) or homologous recombination (HR), resulting in targeted mutations. Currently four families of engineered nucleases are available – meganucleases, ZFNs, TALENs, and the CRISPR/Cas9 technology. The CRISPR/Cas system has surpassed the existing GE tools like ZFNs and TALENs through its simplicity, cost-effectiveness and efficient nature and the era of GE has soared high which is evident with increasing reports of genome editing in crop plants. Very recently, Jung and Altpeter (2016) reported a TALEN mediated genome editing approach in sugarcane and demonstrated the effective targeted mutagenesis to modify the cell wall characteristics for increased production of lignocellulosic ethanol in complex polyploids like sugarcane. However, as far as sugarcane is considered, there are still several challenges ahead that need to be resolved to achieve a complete exploitation of the crop through biotechnological tools. This review describes the various difficulties, issues and regulatory concerns with regard to genome engineering in sugarcane.

## The CRISPR-Cas9 System

Genome editing has become way easier with the introduction of CRISPR/Cas9 technique to engineer plant genomes with desired traits. The technique has been successfully demonstrated with model plants like *Arabidopsis thaliana. Nicotiana tabacum* as well as monocot species such as rice, sorghum, wheat and maize (Jiang et al., 2013; Mao et al., 2013; Gao et al., 2014; Liang et al., 2014; Wang et al., 2014; Zhang et al., 2014). The approach helps us to edit single to multiple genes by knocking-in or knocking-out of a host genome in an efficient manner which has facilitated rapid advancements in genome engineering. It induces double stranded breaks (DSBs) in the genome which are then repaired by either NHEJ or HR thereby leading to mutations in the target site. However, NHEJ is more efficient than HR to create mutant alleles using CRISPR technology (Paul and Qi, 2016). Recently, Shan et al. (2014) have reported a simple and efficient protocol for genome editing in rice and wheat which describes a stepwise approach including exhaustive details on design, construction, verification and use of gRNAs for sequence-specific CRISPR/Cas-mediated mutagenesis and gene targeting.

Though all the three genome editing techniques are used widely, CRISPR method is advantageous owing to the following attributes. Firstly, CRISPR/Cas system depends on basic RNA/DNA hybrids to determine the sequence specificity unlike other methods which solely depend on protein based recognition. The 20 nucleotide sequence present in the gRNA determines specificity by the protospacer adjacent motif (PAM) motif (NGG consensus) and the cleavage is done by Cas9 enzyme. The second important advantage is the possibility to edit multiple genes simultaneously or multiplexing as it is commonly known, using this method which drastically reduces time. Thirdly, both ZFNs and TALENs function as dimers, the vector construction and plant transformation processes are very complicated whereas CRISPR is simple and efficient. Bortesi and Fischer (2015) have highlighted the effectiveness of CRISPR/Cas9 systems and also compared their strengths and weaknesses with the predecessors, the ZFNs and TALENs. Since CRISPR mediated GE in crop plants have been extensively reviewed (Bortesi and Fischer, 2015; Belhaj et al., 2015; Xiong et al., 2015; Cardi and Stewart, 2016; Song et al., 2016), this review is confined to the advantages of GE in a complex polyploid and the various challenges that hamper its improvement through genome engineering.

## Advantages of Genome Editing in Sugarcane

Introduction of a desired trait in a commercial elite variety through traditional breeding in crops with complex genomes like sugarcane is extremely laborious and time consuming. It typically takes 12–15 years to release an improved variety through conventional breeding strategy (Shanthi et al., 2008; Gazaffi et al., 2010). Furthermore, introgression of multiple traits or modifying metabolic pathways is almost impossible. However, with the advent of transgenic technology this feat could be achieved to a certain extent. Now, with the genome editing techniques, these can be accomplished with substantial success which is one of the greatest merits of the GE approach. Using CRISPR technology, not only gene addition but gene elimination (either by random deletion during NHEJ or by gene inactivation by change of sequence or both) is also possible. For instance, one can eliminate the gene responsible for disease susceptibility and make the susceptible variety into a resistant genotype. Wang et al. (2014) used TALEN mediated approach to knock out all three mildew resistant locus (MLO) homoeoalleles and CRISPR technology was employed to develop transgenic wheat plants that have mutations in *TaMLO-A1* allele. Mutations of all three MLO conferred broad spectrum resistance to powdery mildew.

Using CRISPR system, it is possible to edit several genes simultaneously by the introduction of DSBs at several sites (Li et al., 2013; Mao et al., 2013). The ability to stack multiple genes using CRISPR/Cas9 system is also feasible which has a great application in metabolic engineering and molecular farming approaches. Finally, there is a hope that genome edited events would not be classified to be GM (Hartung and Schiemann, 2014) and may have a different regulatory policy that has inevitably increased researchers to generate crops with desirable traits using CRISPR technology. Furthermore, the technology can also be employed to modify desired gene using HR, modify transcriptional regulation and to create site-directed structural changes and thus has unlimited applications in engineering plants.

## The Complexities In A Polyploid Genome and Challenges Ahead

The genome size of sugarcane is about 10 GB with homologous genes ranging from 8 to 12 copies (Souza et al., 2011) and the monoploid genome size being 750–930 MB (D’Hont and Glaszmann, 2001). With the sorghum genome deciphered (Paterson et al., 2009), there have been enhanced genomic studies of its close relatives like sugarcane. Sorghum and sugarcane sequences are well conserved at the gene level, with 85% similarity among the orthologs (Wang et al., 2010). The challenges that sugarcane introduces to the computational biologists and the sequencing consortiums thereby making the whole genome sequencing of sugarcane one of the hardest *de novo* assembly are (i) polyploidy -80% of sugarcane genome is supposed to be inherited from *S. officinarum* and 10% is from *S. spontaneum*; (ii) high levels of recombination – results in the remaining 10% of genome which is derived from both the progenitor species; (iii) heterozygosity - lead to varying degrees of uncertainty during genome assembly (Henry and Kole, 2010); (iv) repeats - polyploidy increases the repeats leading to irregularity. Thus, the large and complex genome, high ploidy and high content of repetitive DNA make sugarcane unusually recalcitrant for both forward as well as reverse genetic studies.

One of the critical requirements for genome editing is the availability of functional genomics resources in order to design specific gRNAs and thereby target specific genes whose function is known. Researchers working in polyploid crops such as sugarcane require knowledge about the sequence variation present between different allelic forms in order to design precise gRNAs. However, the sugarcane genome is not deciphered yet and the genomic resources are limited in sugarcane despite few transcriptomic and EST data. In addition, the functions of over 10,000 sugarcane coding genes are yet to be deciphered (Vicentini et al., 2012).

Transgene silencing is a major drawback that deters molecular improvement in sugarcane. Both transcriptional and post transcriptional transgene silencing effects have been reported (Hansom et al., 1999). Birch et al. (2010) showed that transgene silencing in sugarcane is efficient in the primary transformants, copy number independent, 5′ sequence specific, developmentally regulated and is initially post transcriptional in To plants. Their results also demonstrated that post transcriptional silencing in sugarcane was promoter-cassette sequence specific which reiterates the use of different and efficient promoters in order to alleviate silencing effects. In sugarcane, the maize ubiquitin promoter has been the promoter of choice. Moreover, sugarcane derived promoters have also been subjected to efficient reporter gene silencing (Wei et al., 2003; Mudge et al., 2009). Hence, there is a primary need for the use of specific Cas9 genes regulated by potential high efficient promoters so as to achieve significant editing efficiency and thereby crop improvement.

Like the other genome editing tools, CRISPR/Cas9 system also has the setback of off-target effects which, at some instances, may result in random unnecessary mutations and abnormalities. Cas9:gRNA complexes can cleave target DNA sequences even with several mismatches in the guide sequence, implying that they are capable of cutting at other genomic sites (Fu et al., 2014). Very recently, Osakabe et al. (2016) employed a truncated gRNA/Cas9 combination regulated by a constitutive promoter in *Arabidopsis* with negligible off-target effects and high mutation rates. This method needs to be explored further for usage in GE of sugarcane. Kleinstiver et al. (2016) reported novel modified Cas9 variants by substituting 3–4 amino acids, which could also be exploited to address the off-target problems in plants.

CRISPR/Cas9 technology uses a range of transformation methods like protoplast transfection, agro-infiltration and generation of stable transgenics. Unlike other dicotyledonous species, transient systems in sugarcane such as agro-infiltration or protoplast fusion methods are not so successful. *Agrobacterium* mediated sugarcane transformation, though well established and routinely used, is a time consuming laborious process and has low transformation efficiency (Joyce et al., 2010). The time taken from DNA delivery till whole plant regeneration is longer than the other crop plants which hinder genome engineering process that requires screening of large number of transgenics to identify mutants in a short time. Recently, Lowe et al. (2016) reported an efficient monocot transformation approach wherein they over-expressed the maize morphogenic regulators *Baby boom* (*Bbm*) and *Wuschel2* (*Wus2*) genes in previously non-transformable maize inbred lines and achieved high transformation frequencies. They also demonstrated enhanced transformation in sorghum, sugarcane and rice proving the utility of this approach in monocots.

Yet another demerit is the need for a large number of mutants required for the functional studies of multiple allelic gene forms, typical to polyploids like sugarcane. Unfortunately, there is a huge lack of mutant studies in sugarcane and generating large number of mutants is time consuming and laborious, especially for the genes which are closely linked. However, this can be accomplished using multiple gRNAs, a strategy which was successful in rice (Ma et al., 2015) and wheat (Wang et al., 2016). In sugarcane, most traits are polygenic and hence there is a lack of readily available targets. Tools indispensable to target one specific copy or several homologous copies are inevitable for successful GE in sugarcane.

Another striking challenge ahead is the lack of high throughput screening techniques to identify transgenic plants with gene edited events. Routinely used techniques include PCR, restriction digestion, surveyor assays followed by sequencing by Sanger/NGS. However, these methods are expensive, insensitive, laborious and time consuming. Recently, capillary electrophoretic technique was adopted to identify and characterize the TALEN mediated mutations in the genome edited events and was validated using pyrosequencing (Jung and Altpeter, 2016). It was found that the high throughput method was inexpensive and highly sensitive. However, the use of this method for screening CRISPR based mutants has to be explored further.

Lastly, the challenge raised on the regulatory issues regarding GE approach is worth mentioning. Transgenes encoding Cas9 and/or other editing molecules need to be segregated from edited loci in order to warrant a non-GM status. However, it is not clear whether and how cultivars carrying such transgenes would be labeled as non-GM. Elite sugarcane cultivars targeted for genetic modification are typically derived from highly polyploid and interspecific hybrids and these elite genetic backgrounds would also be concomitantly lost with genome editing transgene segregation which may cause serious implications for breeding strategies aimed at exploiting edited loci. Araki and Ishii (2015) have devised a regulatory policy that classifies the genome edited organisms into product based and process based regulation. Countries like the United States and Canada have accepted the product based regulation whereas Brazil, European Union and other nations went for the process based system. Recently, Huang et al. (2016) have proposed a regulatory framework for precision breeding with genome edited crops that would be beneficial, if considered and adopted. Genome editing technique has also raised serious concerns in terms of ecological imbalance, intellectual property right issues and consumer regulations which might be resolved in the near future. Nevertheless, recent years have established the significance of CRISPR technology which has been a very quickly adopted methodology and the remarkable achievements that researchers have obtained in crop plants are not to be ignored.

## Conclusion and Future Perspectives

Though challenges to be resolved are many, the CRISPR/Cas9 system will undoubtedly evolve into an invincible strategy for precision crop breeding and biotechnology in the near future. As far as sugarcane is considered, we hope that with the availability of the whole genome, remarkable advances would be feasible in terms of genome engineering in sugarcane to generate improved new varieties with specific desirable traits. Furthermore, availability of advanced bioinformatic tools that aid in trait specific gRNA design and high throughput screening techniques to identify mutants will undoubtedly lead the GE platform into new horizons to create designer crops in general and sugarcane in particular, that would benefit the human population. An appropriate regulatory policy that distinguishes between GMOs and GE organisms will enable us to exploit these techniques in an efficient manner.

## Author Contributions

CM conceived and wrote the manuscript.

## Conflict of Interest Statement

The author declares that the research was conducted in the absence of any commercial or financial relationships that could be construed as a potential conflict of interest.
